# Supplementary material for: Impact of Variants in the ATIC and ARID5B Genes on Therapeutic Failure with Imatinib in Patients with Chronic Myeloid Leukemia
Source: Genes (Basel). 2022 Feb 10;13(2):330. doi: 10.3390/genes13020330 (PMC8872593; doi:10.3390/genes13020330)
Supplement: Supplementary file 1 [file genes-13-00330-s001.zip › genes-1515643-SI.pdf]

Table S1: Polymorphisms chosen after applying the criteria of selection

Gene	SNP	Allele	Function	Amino acid substitution	Chromosome	Location
ABCC1	rs28364006	A>G	Missense	Thr1337Ala	16	16134392
ABCC2	rs717620	C>T	5' UTR	-	10	99782821
ABCC3	rs9895420	T>A	5' Flanking	-	17	50634677
AMPD1	rs17602729	G>A	Stop Codon	Gln45Ter	1	114693436
ARID5B	rs10821936	C>T	Intronic	-	10	61963818
ATIC	rs2372536	C>G	Missense	Thr116Ser	2	215325297
ATIC	rs4673993	T>C	Splicing region	-	2	215347616
CCND1	rs9344	G>A	Synonym	Pro241Pro	11	69648142
CDKN2A	rs3731217	A>C	Intra-genic	-	9	21984662
CEBPE	rs2239633	G>A	5' UTR	-	14	23119848
GGH	rs11545078	G>A	Missense	Thr151Ile	8	63026205
GGH	rs1800909	A>G	Missense	Cys6Arg	8	63038753
GGH	rs3758149	G>A	5' Flanking	-	8	63039169
IKZF1	rs4132601	T>G	UTR '3	-	7	50402906
ITPA	rs1127354	C>A	Missense	Pro15Thr	20	3213196
MTHFD1	rs2236225	G>A	Missense	Arg653Gln	14	64442127
MTHFR	rs1801133	G>A	Missense	Ala222Val	1	11796321
MTRR	rs1801394	A>G	Missense	Ile22Met	5	7870860
NALCN	rs7992226	A>G	Intronic	-	13	101145489
NOS3	rs1799983	T>G	Missense	Asp298Glu	7	150999023

PIP4K2A	rs7088318	C>A	Intra-genic	-	10	22564019
SHMT1	rs1979277	G>A	Missense	Leu435Phe	17	18328782
SLCO1B1	rs2306283	A>G	Missense	Asn130Asp	12	21176804
SLCO1B1	rs4149015	G>A	5' Flanking	-	12	21130388
SLCO1B1	rs4149056	T>C	Missense	Val174Ala	12	21178615
TLR4	rs4986790	A>G	Missense	Asp99Gly	9	117713024
TNFAIP3	rs6920220	G>A	Intra-genic	-	6	137685367
TPMT	rs1800460	C>T	Missense	Ala154Thr	6	18138997
TPMT	rs1800462	C>G	Missense	Ala80Pro	6	18143724
TPMT	rs1142345	T>C	Missense	Tyr240Cys	6	18130687
TPMT	rs12201199	A>T	Intrônico	-	6	18139571
TPMT	rs56161402	C>T	Missense	Arg215His	6	18130762

3UTR: 3’UTR regulation; 5UTR: 5’UTR regulation

Table S2: Allelic and genotypic distribution and quality control of the polymorphisms.

Gene/rs	Most Frequent Allele	Nucleotide change	Missings[5]	AA[1]	Aa[2]	aa[3]	Missings (%)[4]	MAF[5]
TPMT rs1142345	T	T>C	42	90	30	6	25.0	16.7
TPMT rs12201199	A	T>C / T>G	35	95	35	3	20.8	15.4
SLCO1B1 rs4149056	T	T>C	31	94	38	5	18.5	17.5
ABCC2 rs717620	C	C>T	29	112	25	2	17.3	10.4
ABCC3 rs9895420	T	T>A	27	111	29	1	16.1	11.0
GGH rs11545078	G	G>A	21	114	30	3	12.5	12.2
GGH rs3758149	G	G>A	35	66	58	9	20.8	28.6
ATIC rs2372536	C	C>G	25	81	51	11	14.9	25.5
ATIC rs4673993	T	T>C	25	75	55	13	14.9	28.3
AMPD1 rs17602729	G	G>A	35	114	17	2	20.8	7.9
CCND1 rs9344	G	G>A	18	55	73	22	10.7	39.0
IKZF1 rs4132601	T	T>G	26	90	46	6	15.5	20.4
ITPA rs1127354	C	C>A	44	114	10	-	26.2	4.0
MTRR rs1801394	G	A>G	41	47	61	19	24.4	39.0
MTHFD1 rs2236225	G	G>A	31	32	74	31	18.5	49.6
NOS3 rs1799983	G	T>G	44	121	3	-	26.2	1.2
MTHFR rs1801133	G	G>A	18	69	66	15	10.7	32.0
TLR4 rs4986790	A	A>G	31	129	8	-	18.5	2.9
TPMT rs1800460	C	C>T	24/48	127	13	4	14.3	7.3
SLCO1B1 rs4149015	G	G>A	82	73	9	4	48.8	9.9
GGH rs1800909	A	A>G	27	132	5	4	16.1	4.6
ARID5B rs10821936	T	C>T	26	42	59	41	15.5	49.6
NALCN rs7992226	A	A>G	46	50	47	25	27.4	39.8
SHMT1 rs1979277	A	G>A	67	63	27	11	39.9	24.3
SLCO1B1 rs2306283	G	A>G	52	35	59	22	31.0	44.4
CEBPE rs2239633	G	G>A	53	50	53	12	31.5	33.5
PIP4K2A rs7088318	A	C>A	53	31	67	17	31.5	43.9
TNFAIP3 rs6920220	G	G>A	58	79	30	1	34.5	14.5
TPMT rs1800462	C	C>G	28	140	-	-	16.7	0.0
TPMT rs56161402	C	C>T	33	270	-	-	19.6	0.0
ABCC1 rs28364006	A	A>G	15	153	-	-	8.9	0.0
CDKN2A rs3731217	A	A>C	47	121	-	-	28.0	0.0

[1]AA: Most frequent homozygote; [2]Aa: Heterozygote; [3]aa: Less frequent homozygote; [4]Genotyping data not obtained; [5]MAF: minor frequency allele

Table S3. Odds ratio and genotype distributions of the polymorphisms not statistically significant between the patients with response and without response.

Genotype	Responders (%)	No responders (%)	OR (95% CI)	Lower	Upper	p-value
TPMT rs1142345						
TT	57 (71.2)	32 (71.1)	1.00			0.25
CT	21 (26.2)	9 (20.0)	0.76	0.31	1.86	
CC	2 (2.5)	4 (8.9)	3.56	0.62	20.54	
TPMT rs12201199						
AA	54 (67.5)	40 (78.4)	1.00			0.15
AT	25 (31.2)	9 (17.6)	0.49	0.2	1.15	
TT	1 (1.2)	2 (3.9)	2.7	0.24	30.82	
SLC01B1 rs414956						
TT	61 (74.4)	33 (62.3)	1.00			0.30
CT	18 (22.0)	18 (34.0)	1.85	0.85	4.03	
CC	3 (3.7)	2 (3.8)	1.23	0.2	7.75	
ABCC2 rs717620						
CC	66 (80.5)	44 (81.5)	1.00			0.93
CT	15 (18.3)	9 (16.7)	0.9	0.36	2.24	
TT	1 (1.2)	1 (1.9)	1.5	0.09	24.62	
ABCC3 rs9895420						
TT	68 (81.0)	41 (75.9)	1.00			0.63
AT	15 (17.9)	13 (24.1)	1.44	0.62	3.32	
AA	1 (1.2)	0 (0.0)	0.00	0.00		
GGH rs11545078						
GG	68 (78.2)	43 (75.4)	1.00			0.88
AG	17 (19.5)	13 (22.8)	1.21	0.53	2.74	
AA	2 (2.3)	1 (1.8)	0.79	0.07	8.99	
GGH rs3758149						
GG	40 (50.6)	24 (47.1)	1.00			0.59
AG	35 (44.3)	22 (43.1)	1.05	0.5	2.19	
AA	4 (5.1)	5 (2.08)	2.08	0.51	8.52	
ATIC rs2372536						
CC	51 (60.0)	29 (52.7)	1.00			0.22
CG	30 (35.3)	19 (34.5)	1.11	0.53	2.32	
GG	4 (4.7)	7 (12.7)	3.08	0.83	11.41	
ATIC rs4673993						
TT	49 (57.0)	25 (46.3)	1.00			0.33
CT	31 (36.0)	22 (40.7)	1.39	0.67	2.88	
CC	6 (7.0)	7 (13.0)	2.29	0.69	7.53	
AMPD1 rs17602729						
GG	71 (87.7)	41 (82.0)	1.00			0.38
AG	8 (9.9)	9 (18.0)	1.95	0.70	5.44	

AA	2 (2.5)	0 (0.0)	0.00	0.00		
CCND rs9344						
GG	35 (38.5)	20 (35.7)	1.00			0.93
AG	43 (47.3)	27 (48.2)	1.10	0.53	2.28	
AA	13 (14.3)	9 (16.1)	1.21	0.44	3.33	
IKZF1 rs4132601						
TT	57 (66.3)	32 (60.4)	1.00			0.43
GT	25 (29.1)	20 (37.7)	1.42	0.69	2.96	
GG	4 (4.7)	1 (1.9)	0.45	0.05	4.16	
ITPA rs1127354						
CC	71 (93.4)	41 (89.1)	1.00			0.40
AC	5 (6.6)	5 (10.9)	1.73	0.47	6.34	
MTRR rs1801394						
AA	26 (32.9)	20 (42.6)	1.00			0.52
AG	41 (51.9)	20 (42.6)	0.63	0.29	1.4	
GG	12 (15.2)	7 (14.9)	0.76	0.25	2.28	
MTHFD1 rs2236225						
GG	19 (22.6)	13 (25.5)	1.00			0.79
AG	48 (57.1)	26 (51.0)	0.79	0.34	1.86	
AA	17 (20.2)	12 (23.5)	1.03	0.37	2.87	
NOS3 rs1799983						
GG	76 (98.7)	43 (95.6)	1.00			0.29
GT	1 (1.3)	2 (4.4)	3.53	0.31	40.13	
MTHFR rs1801133						
GG	44 (48.9)	25 (43.9)	1.00			0.50
GA	40 (44.4)	25 (43.9)	1.10	0.55	2.22	
AA	6 (6.7)	7 (12.3)	2.05	0.62	6.79	
TLR4 rs4986790						
AA	77 (92.8)	50 (96.2)	1.00			0.40
AG	6 (7.2)	2 (3.8)	0.51	0.1	2.64	
TPMT rs1800460						0.05
CC	77 (89.5)	47(85.5)	1.00			
CT	9 (10.5)	4 (7.3)	0.73	0.21	2.50	
TT	0 (0)	4 (7.3)		0.00		
GGH rs1800909						0.35
AA	79 (95.2)	50 (90.9)	1.00			
AG	3 (3.6)	2 (3.6)	1.05	0.17	6.53	
GG	1 (1.2)	3 (5.5)	4.74	0.48	46.84	
ARID5B rs10821936						0.30
TT	22 (25.9)	20(37.0)	1.00			
CT	36 (42.4)	22 (40.7)	0.67	0.30	1.50	
CC	27 (31.8)	12 (22.2)	0.49	0.20	1.22	
NALCN rs7992226						0.38
AA	27 (37.5)	21 (43.8)	1.00			

AG	27 (37.5)	20 (41.7)	0.95	0.42	2.15
GG	18 (25.0)	7 (14.6)	0.50	0.18	1.42
